# Medical Gleanings from the American Arctic Expedition

**Published:** 1852-03

**Authors:** 


### ECLECTIC DEPARTMENT,

### AND SPIRIT OF THE MEDICAL PERIODICAL PRESS.

### *Medical Gleanings from the American Arctic Expedition.* By BENJAMIN VREELAND, M. D., Assistant Surgeon U. S. Navy.

We sailed from New York on the 23d of May, 1850, with a crew that had been promiscuously obtained, and whose physical strength was not well adapted to withstand the hardships to be expected on the cruise. Several of the men were even suffering from chronic affections, and but few of them possessed that robustness which the service demanded. The majority of the officers and crews, however, were much nearer twenty than thirty years of age, and had all the endurance and enthusiasm natural to young men. The total number of souls on board both vessels was thirty-five,* of which eight were officers. The vessels were very small, of one hundred and fifty and ninety tons burden respectively, and were actually so loaded down with provisions, that at sea, our decks were constantly washed by water ankle deep, which often poured down the hatches of the cabin and forecastle. The quarters of the officers and men were necessarily confined, and as no fire was placed below until late in the fall, their dampness and bad ventilation caused great inconvenience and discomfort. During the voyage out to Baffin's Bay, almost all suffered from bronchial and rheumatic affections; in one case, a relapse of intermittent fever was undoubtedly brought on by these unfavorable circumstances, and the rawness of the climate produced crops of chilblains, covering the hands and feet, that were exceedingly painful and annoying. We arrived at our first rendezvous, the Whale Islands, in 69° north latitude, on the 27th of June, and after a delay of two days, sailed, and made the packed ice on the 7th of July. From this date to August 16th, when we reached the north water in Baffin's Bay, in latitude 76° north, we were constantly exposed to the numerous sources of disease necessarily attendant upon the tedious and dangerous labor of navigating the vessels through the ice. The ships' companies were worked in watches without intermission, and it not unfrequently happened that all hands were employed for twenty-four hours in succession, in heaving, warping, breaking, and sawing through ice from one to eight feet in thickness; the weather during the time being generally foggy and unpleasant, and the thermometer ranging from 25° to 42° Fah't. While thus engaged, the feet were continually wet, and the clothing damp, from being obliged to wade through pools of melting snow; it was a common accident for individuals to fall overboard, and be entirely immersed in water, the temperature of which never ranged higher than 32°, and frequently was as low as 28°; when an accident of this kind occurred, assistance was immediately required to get the person out, for in a short time he became so benumbed as to be unable to help himself. Fortunately, we had not much sickness while exposed to this cold, wet, and fatiguing labor. The change and drying of wet clothes was required of the men as fast as practicable; but the means were so scant, and opportunities so few, that many were obliged to wear their clothing imperfectly dried; in consequence, colds and rheumatic pains were frequent, and to that cause the early appearance (on the 25th of July) of a case of scurvy was particularly attributed; the young man who was attacked being discovered to be very negligent and careless in that respect. On the 13th September, the temperature falling to 8°, the vessels were frozen in at the entrance to Wellington Channel, in 74° north latitude, and 93° west longitude, and our position was so uncertain and so perilous, owing to the rapid drift and crushing of the ice, that preparations for the winter could not be made, and stoves were not erected until the 19th of October. In the mean time the thermometer had fallen to 11°, and the discomfort experienced at that period surpassed any that was felt during the remaining portion of the winter. It was impossible to keep warm without constant exercise; the vapors arising from our bodies, condensed on the timbers, bulkheads and in our sleeping places, first as water, rendering them very wet and unwholesome, and as the temperature decreased, in the form of ice and snow. The only fire since leaving New York, had been

* Two were sent home from our first rendezvous, leaving thirty-three all told.

kept in the galley on deck, where the necessary cooking operations were performed. No clothing or bedding could be carried on deck for the purpose of ventilation, for, on being returned below, the surrounding vapors would immediately condense upon them, and render them much more wet than before. Metallic bodies brought below from the external atmosphere, would be instantly covered with a sheeting of ice. Scurvy began to appear in earnest on the 28th of September, and continued among us, until our disruption from the ice in the beginning of June, 1851. Disease was to be expected under such circumstances; and that we suffered so little serious sickness as we did, may perhaps be attributed to the constant exercise we were obliged to take, in order to maintain a necessary amount of animal heat. It was not until the 1st of November, that the Advance was ready to receive the officers and crew of the Rescue for the winter. Every thing was done that could render the vessel as comfortable as she possibly could be. The stores were taken from the hold, and placed on the decks of the Rescue, and the whole interior was open and unconfined by partition or bulkhead. The galley was placed on the kelson amidships; forward of which, were the men's quarters, in which was a stove, and aft, the officers', where another stove was situated. These three fires were quite sufficient to give us a comfortable heat during the most intense cold of winter; the thermometer placed near the center of the vessel, averaging about 60°. This temperature prevented all condensation in the open parts of the vessel, but in the lockers and on the metallic fastenings at the sides, water and ice was continually forming. A constant and effectual ventilation, although absolutely necessary, was impracticable, but in order that there might be some escape for the impurities generated below, the cabin hatch was always kept open; notwithstanding which, we were obliged to breathe lamp smoke and coal ashes during the whole winter, and in such quantity that we never expectorated, without bringing up these substances in great abundance. The sun left us on the 7th of November, and did not appear again until the 28th January, a period of eighty-two days. After the sun had set, cases of scurvy increased rapidly, but the symptoms never progressed so far as to produce any serious apprehensions. Not a man was ever confined to his bed, and although some were lame, and unable to use one lower extremity, they were always, during the time appropriated to exercise, compelled to take a certain quantity in company with their messmates.

The causes which seemed to have a direct influence in producing and prolonging the cases under our observation were, the long absence of solar light, a diet without change or variety, want of a proper exciting exercise, personal uncleanliness, dreary monotony, and consequent depression of spirits. The symptoms were generally uniform, most always the first change noticed being a peculiar white arch on the gums, at the root of one or more of the incisor teeth in either jaw, followed in a few days by sponginess, lividity, ulceration, and bleeding. Subsequently, the lower extremities would become painful, swollen, indurated, and discolored by ecchymosis. Sometimes the legs would have the appearances above described, at the same time that the gums continued perfectly healthy. Irritation of the rectum, with frequent small, slimy, and bloody stools, accompanied by pain and tenesmus, was also frequent. In December, our boatswain's mate, about fifty years of age, the oldest man in the vessels, was seized with pneumonia, which at one time seemed likely to prove fatal, but he gradually recovered, and during convalescence suffered severely from ulceration and loosening of the gums, brought on by his long confinement below. We had but one severe case of frost-bite, in which a part of the helix of the ear sloughed away. Superficial frost-bites, however, causing vesication of the fingers, nose, ears, and cheeks, were continually occurring. If the slightest wind was stirring, the ends of the nose and the lobes of the ears of some of us would freeze, and we would remain unconscious of the fact until informed by a companion. In the spring several of the officers and men were rendered snow blind by the peculiar glare of the snow which exists in overcast weather. On bright, sunshiny days, we walked on the dazzling ice and snow with impunity, but when the sky was at all obscured by clouds, the light reflected from the snow was such as to deceive us as to the true distance and size of objects; and the unevenness of the surface of the ice was so disguised, that we were unable to tell an elevation from a depression; consequently, we would step off from pieces of ice three or four feet high, without being conscious of any change of surface, until we found ourselves falling, and again, we would trip over inequalities that were insensible to us until it was too late to raise our feet high enough to clear them. This indistinctness and uncertainty of vision, brought on a very acute conjunctivitus, that for thirty-six or forty-eight hours, was very painful. The most grateful application was cold water, and in four or five days, the eyes were apparently as well as ever. The relations between the officers and men were of the most easy and pleasant nature, privations and hardships were shared alike by all, and the few comforts we were possessed of, equally distributed and enjoyed. The discipline practiced during the winter had direct reference to the preservation of the vessel, and the health of the crew; and was so apparent to every man, that its importance was always appreciated. But little difficulty was experienced in enforcing obedience, and a murmur or complaint was rarely heard. The daily ration for each man, for four days in the week, consisted of one pound of fresh beef or mutton, and three-quarters of a pound of preserved vegetables, either potatoes, carrots or beets. On the alternate three days, one pound of salt pork or beef was issued, and, in addition, all the other articles of the navy ration. A liberal supply of vinegar, pickles, and preserved cranberries was allowed, and an abundance of well fermented bread was made daily. The fresh provisions had been previously cooked, and preserved in tin canisters, hermetically sealed, and at first were quite palatable, but in a short time, they became insipid and tasteless, and toward the close of the winter, such was the disrelish and disgust for them, that not one-half of the ration was eaten. A little variety, in the way of bears' and foxes' flesh, was now and then obtained, and enjoyed exceedingly. They were considered luxuries, and mostly appropriated to the scorbutic, upon whom they seemed to have an excellent effect, merely by the change in diet, which they afforded. The men's gums and shins were daily examined, and their personal cleanliness strictly inspected, as also the condition of their apartment, in regard to its dryness, cleanliness, &c. At the same time from two to four ounces of lime juice was made into lemonade, and taken by each man in the presence of one of the medical officers. The officers generally drank about the same quantity at dinner. Daily exercise was one of the most important duties to be attended to, and as there was always sufficient light for about four hours in the middle of the day, to enable us to walk a mile or two from the vessel, and play at various games, such as foot-ball, skating, sliding, &c., every man was required to engage in them. On board, reading was the chief occupation, and when this grew wearisome, cards and games of all kinds were resorted to, to relieve ennui; and once every fortnight, theatrical entertainments were given by the crew, under the encouragement and zealous assistance of the officers. The latter amusements were well adapted to enliven the sailors, for they gave pleasant excitement and employment for days in succession, in preparing *roles*, and in manufacturing dresses, scenery, &c. It may be a sufficient proof of the interest which these plays created, and the infinite amusement they afforded, to say, that on the very coldest night of the winter, we sat on deck, viewing and applauding representations in which female characters appeared on the stage, with bare necks and arms, when the thermometer was 46° below zero. Towards the end of the long nights, a loss of flesh and strength was observed in all of us; we had become bleached to a pale, waxy color, and our hair came out abundantly. The anti-scorbutics, in a measure, lost their effect, and possessed but the power of holding the disease in check, for symptoms did not begin to disappear and cases to recover permanently until after the rising of the sun; the exhilaration excited by his re-appearance seemed to have a direct and beneficial influence. Out of the whole complement, one officer and nineteen men had unequivocal symptoms of scurvy. The remaining seven officers and seven men enjoyed comparatively good health during the whole cruise. The disruption of the ice on the 5th of June, in north latitude 66° 32′, and west longitude 59° 40′, liberated the vessels, and with all possible dispatch, we made the first convenient settlement on the coast of Greenland. Here we obtained fresh fish, seals, and scurvy grass, by which the health of the ships' companies was recruited, and their strength partly restored. But we felt conscious, and it was evident from our altered appearance, that we did not possess the vigor of the preceding year, and the probability is, that if we had been detained another winter in those regions, a number of our party would not have survived it. We then proceeded north again, with the intention of continuing the search, but being unable to penetrate the ice in the northern part of Baffin's Bay, and having waited for an opening until the 18th of August, we made sail for home, where we arrived on the 7th of October.

